# Exploring root system architecture and anatomical variability in alfalfa (*Medicago sativa* L.) seedlings

**DOI:** 10.1186/s12870-023-04469-4

**Published:** 2023-09-25

**Authors:** Xinya Pan, Pengfei Wang, Xianwei Wei, Jinxin Zhang, Bingcheng Xu, Yinglong Chen, Gehong Wei, Zhi Wang

**Affiliations:** 1 College of Grassland Agriculture, Northwest A&F University, 3 Taicheng Road, Yangling, 712100 China; 2 State Key Laboratory of Soil Erosion and Dryland Farming on the Loess Plateau, Northwest A&F University, 26 Xinong Road, Yangling, 712100 China; 3 The UWA Institute of Agriculture, School of Agriculture and Environment, The University of Western Australia, Perth, WA 6001 Australia; 4 College of Life Sciences, Northwest A&F University, 22 Xinong Road, Yangling, 712100 China

**Keywords:** Alfalfa, Root system architecture, Root anatomical traits, Variability, Visual rhizobox

## Abstract

**Background:**

The growth of alfalfa (*Medicago sativa* L.) is significantly hampered by drought and nutrient deficiencies. The identification of root architectural and anatomical characteristics holds paramount importance for the development of alfalfa genotypes with enhanced adaptation to adverse environmental conditions. In this study, we employed a visual rhizobox system to investigate the variability in root system architecture (including root depth, root length, root tips number, etc.), anatomical features (such as cortical traits, total stele area, number and area of vessel, etc.), as well as nitrogen and phosphorus uptake across 53 alfalfa genotypes during the seedling stage.

**Results:**

Out of the 42 traits measured, 21 root traits, along with nitrogen (N) and phosphorus (P) uptake, displayed higher coefficients of variation (CVs ≥ 0.25) among the tested genotypes. Local root morphological and anatomical traits exhibited more significant variation than global root traits. Twenty-three traits with CVs ≥ 0.25 constituted to six principal components (eigenvalues > 1), collectively accounting for 88.0% of the overall genotypic variation. Traits such as total root length, number of root tips, maximal root depth, and others exhibited positive correlations with shoot dry mass and root dry mass. Additionally, total stele area and xylem vessel area showed positive correlations with N and P uptake.

**Conclusions:**

These root traits, which have demonstrated associations with biomass and nutrient uptake, may be considered for the breeding of alfalfa genotypes that possess efficient resource absorption and increased adaptability to abiotic stress, following validation during the entire growth period in the field.

**Supplementary Information:**

The online version contains supplementary material available at 10.1186/s12870-023-04469-4.

## Background

Alfalfa (*Medicago sativa* L.) is one of the most important fodder crops in the world, with rich protein concentration, strong biological nitrogen fixation ability, wide adaptability and high yield [1]. The rapid development of the livestock industry has strongly increased the demand for alfalfa in developing countries such as China [2]. However, the growth of alfalfa is largely limited by abiotic stresses such as nutrient limitations and drought stress [3]. The root system determines the ability to forage soil resources and plays an irreplaceable role in plant growth and adaptability [4]. Selecting crop genotypes with desirable root systems is essential to improve the absorption capacity of soil resources and the adaptability to environmental stresses [5].

Root system architecture (RSA) refers to the shape (mainly including morphology and topological structure) and spatial distribution of the root system in the growth medium [6]. Altering RSA is a key adaptation strategy for crops to cope with edaphic stresses such as drought and nutrient deficiency [7]. Among root morphological traits, total root length is an important trait closely related to root mass, root depth and absorptive capacity and also reflects the size of the root system [8–10]. The large root system can promote soil resource uptake and leaf development [11, 12]. Under drought stress, maize increases its rooting depth to enhance the absorption of water from deep soil [13]. Higher volume, width and number of roots are key traits for alfalfa to acquire water efficiently under water-limited conditions [14]. Two alfalfa genotypes, Arkaxiya and Longzhong, show an increase in specific root length in response to phosphorus deficiency [15]. Higher specific root length increases the extent of root-soil exploration that contributes to phosphorus acquisition under phosphorus-limited conditions [16]. Few studies on the root morphology of alfalfa mainly used static and destructive sampling methods, which makes it hard to reflect the real situation of root structure and growth characteristics [14, 17, 18].

Root foraging ability is not only influenced by morphology but also closely related to anatomical characteristics [5]. Root anatomy is the key factor affecting the lateral and vertical transport of water and nutrients within the root system [19, 20]. Fewer cortical cell files reduce the radial transport resistance of nutrients in the root system and improve water capture by reducing the metabolic costs of soil exploration under drought conditions [19, 21]. Changes in the internal structure of roots, such as increasing cortex-to-stele ratio make a better balance between the absorption and transportation of nutrients [22]. Stele diameter and stele diameter to root diameter ratio of absorptive roots are positively correlated with hydraulic conductance and water transpiration in plants [23]. Previous studies have mainly focused on morphologic or anatomic traits in alfalfa root research [17, 24]. Therefore, integrating root anatomical and morphological traits would be beneficial for enhancing the understanding of root strategies to cope with abiotic stresses and breeding alfalfa genotypes with efficient root systems.

The opacity and complexity of the root growth environment make it challenging to monitor and sample the root. As a result, the research progress of the root system lags far behind that of the aboveground [14]. Rhizobox is an efficient method for dynamically (non-invasive in situ) monitoring root growth through a transparent plate [25]. It can make up for the deficiency of destructive sampling of the root system and obtain complete RSA [26]. Root traits of plants have extensive phenotypic and genetic diversity [27]. But the diversity of alfalfa root traits has been poorly investigated and root morphology and anatomy are rarely combined when dissecting the role of roots in alfalfa adaptation [17, 28]. This study investigated the variability in root morphological and anatomical traits among 53 alfalfa genotypes at the seedling stage using the rhizobox technique [19, 28]. We aimed to characterize the variations in root morphological and anatomical traits and reveal the relationship between key root traits and nutrient uptake as well as plant growth. The results may help advance the breeding process of alfalfa based on root traits and provide insight into the role of key root traits in nutrient uptake and growth of alfalfa.

## Results

### Variation in global traits

**Fig. 1:**
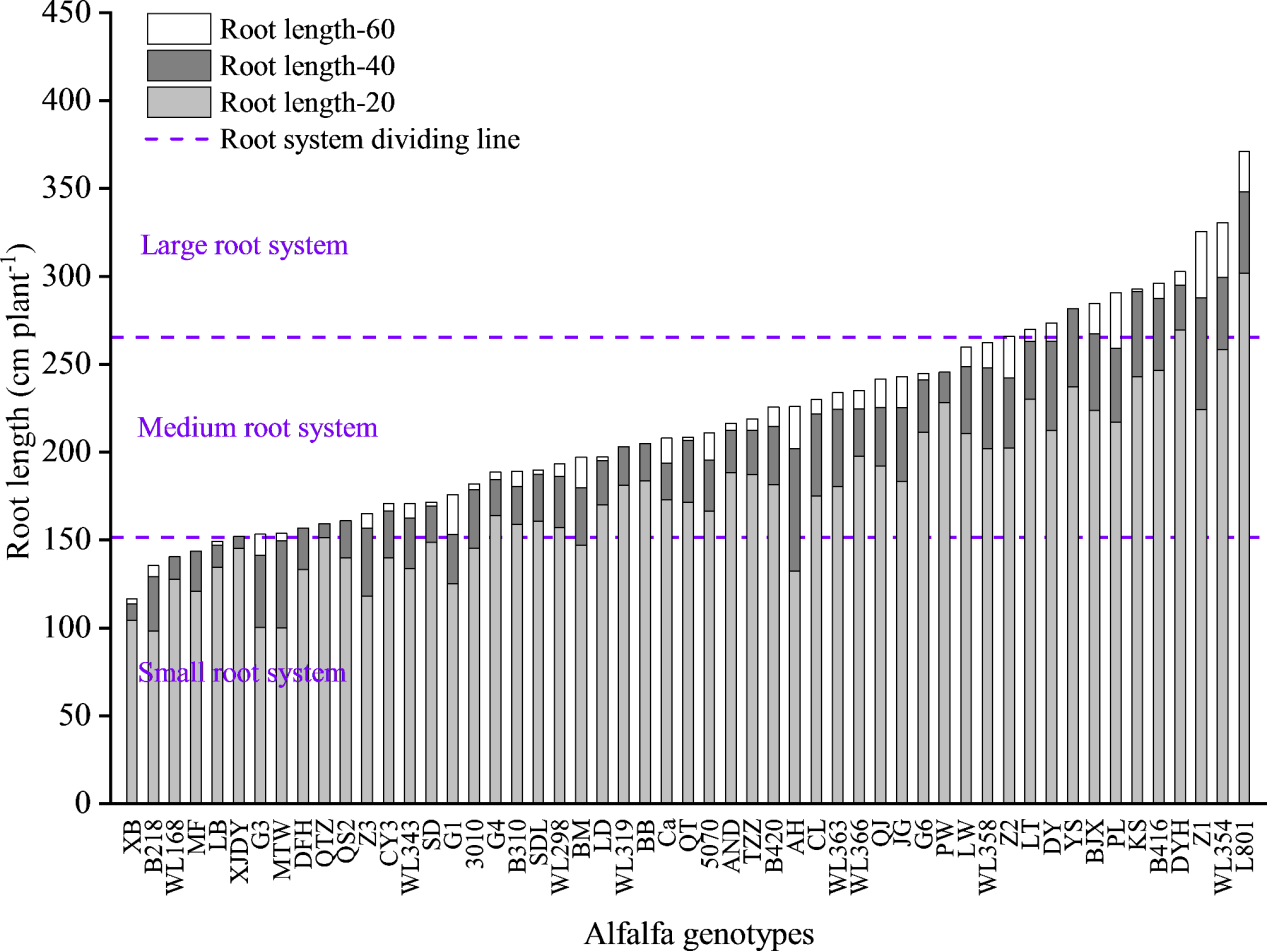
Variation in root length for three groups of alfalfa genotypes presented by root system size. Data were plotted from the lowest to the highest total root length values. Root length-60, root length in 40–60 cm layer; Root length-40, root length in 20–40 cm layer; Root length-20, root length in 0–20 cm layer. The genotypes were classified into small, medium or large root systems according to their total root length per plant. The median value of root length (208.48 cm plant-1) ± standard deviation (57.04 cm plant-1) was used to define the interval for the medium-sized group, and the upper and lower boundaries of the medium interval were constructed by adding to, or subtracting from the median point

**Fig. 2:**
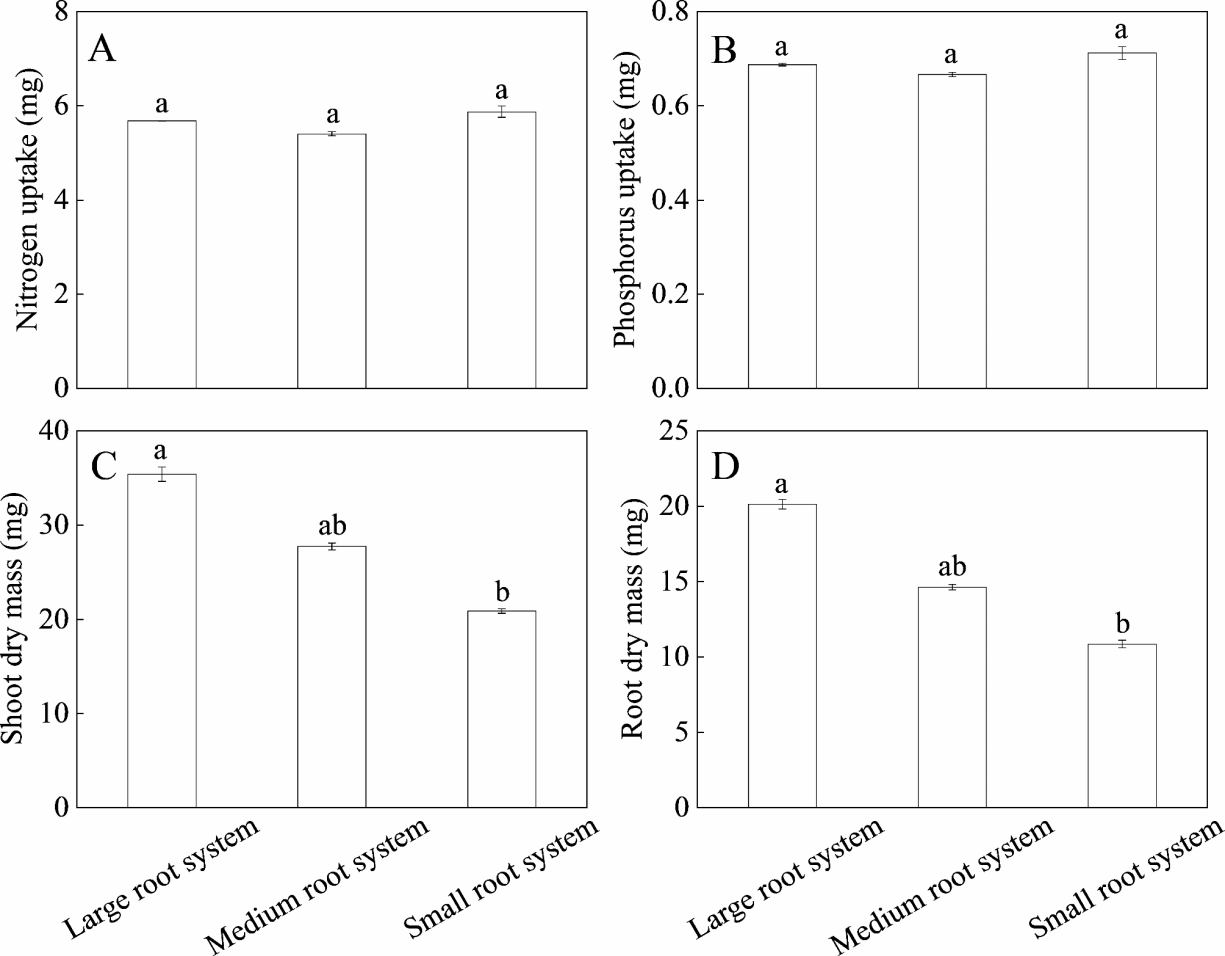
Nitrogen uptake **(A)**, phosphorus uptake **(B)**, shoot dry mass **(C)** and root dry mass **(D)** for three groups of alfalfa genotypes presented by root system size. Different letters indicate significant differences among the three root system size groups (*P* < 0.05)

Among the global traits, the CVs of nitrogen uptake, phosphorus uptake, total root length and root tips number were ≥ 0.25 (Table 1). Total root length ranged from 116 to 371 cm, with an average of 217 cm. The largest genotype had a total root length that was 3.20-fold times greater than the smallest genotype. Twelve genotypes (LT, DY, YS, BJX, PL, KS, B416, DYH, WL354, Z2, Z1 and L801) had large root systems, and 36 genotypes had medium root systems, and 5 genotypes (XB, B218, WL168, MF and LB) had small root systems (Fig. 1). The Chinese breeding line LM801 had the longest total root length and the highest root dry mass among all alfalfa genotypes. Nitrogen uptake, phosphorus uptake, root angle, maximal root depth, root width and branch intensity were significantly different among the tested genotypes (*P* < 0.05). There was no significant difference in nitrogen and phosphorus uptake among the three root system size groups (Fig. 2). The CVs of three shoot traits, including trefoil number, shoot height and shoot dry mass were all < 0.25, and there was no significant difference among tested genotypes. Shoot dry mass and root dry mass of genotypes with large root systems were higher than those of genotypes with medium root systems followed by genotypes with small root systems.

There were significant differences in root spatial distribution (root angle, maximal root depth and root width) among tested alfalfa genotypes (*P <* 0.05). Root angles ranged from 106° to 168°, with an average of 140°. Root width ranged from 8.47 to 21.2 cm, with an average of 17.0 cm. And the maximal root depth ranged from 20.2 to 51.8 cm, with an average of 39.1 cm. There were 29 alfalfa genotypes with a maximum root depth of more than 40.0 cm. The introduced cultivars WL354 and QTZ had the maximum and minimum root depth, respectively.

**Table 1:** Descriptive statistics of 42 measured traits (21 global traits, 14 local traits and 7 root anatomical traits) of 53 alfalfa genotypes

Traits	Minimum	Maximum	Mean	Median	SD	CV	*P* value
**Global traits**							
TN	3.00	6.33	4.28	4.33	0.64	0.15	0.202
SH	5.97	12.7	8.77	8.50	1.58	0.18	0.099
NU	1.93	9.77	5.51	5.14	1.89	**0.34**	**0.000**
PU	0.22	1.23	0.68	0.70	0.22	**0.33**	**0.000**
SDM	15.8	50.7	28.8	28.1	6.84	0.24	0.571
RDM	9.00	25.2	15.5	15.4	3.65	0.24	0.276
TDM	25.1	75.9	44.4	45.2	9.93	0.22	0.495
RA	106	168	140	141	13.0	0.09	**0.020**
MRD	20.2	51.8	39.1	40.5	7.60	0.19	**0.007**
RW	8.47	21.2	17.0	17.2	2.92	0.17	**0.037**
RD	0.29	0.36	0.33	0.33	0.02	0.05	0.264
RL	116	371	217	208	57.0	**0.26**	0.073
RSA	12.8	35.3	21.1	20.9	5.09	0.24	0.131
RV	0.10	0.27	0.16	0.17	0.04	0.23	0.231
RTN	34.3	108	70.1	70.7	18.9	**0.27**	0.281
SRL	8.54	19.5	14.4	14.3	1.86	0.13	0.559
SRA	0.89	1.95	1.40	1.40	0.16	0.11	0.747
RSM	0.41	1.23	0.56	0.55	0.12	0.22	0.151
RTD	77.6	240	96.9	94.1	22.4	0.23	0.450
RLI	3.43	8.60	5.83	5.75	1.30	0.22	0.836
BI	0.21	0.51	0.33	0.31	0.06	0.19	**0.010**
**Local traits**							
RL-thin	25.3	157	77.6	73.5	30.4	**0.39**	**0.015**
RL-thick	71.9	214.3	140	144	32.8	0.23	0.204
RTN-20	22.7	72.7	49.6	50.7	13.7	**0.28**	0.773
RTN-40	3.00	27.0	16.2	15.7	6.65	**0.41**	0.271
RTN-60	0.00	17.3	4.32	3.00	4.41	**1.02**	**0.021**
RL-20	98.2	302	176	173	46.5	**0.26**	0.373
RL-40	6.75	69.9	32.0	29.8	13.2	**0.41**	0.107
RL-60	0.00	37.8	9.16	7.37	9.16	**1.00**	**0.035**
RA-20	8.92	28.5	16.8	16.7	4.21	**0.25**	0.466
RA-40	0.86	6.84	3.31	3.24	1.22	**0.37**	0.160
RA-60	0.00	3.29	0.95	0.84	0.87	**0.92**	**0.011**
RV-20	0.06	0.21	0.13	0.13	0.03	**0.25**	0.531
RV-40	0.01	0.05	0.03	0.03	0.01	**0.34**	0.249
RV-60	0.00	0.03	0.01	0.01	0.01	**0.86**	**0.003**
**Anatomical traits**							
TCA	0.11	0.58	0.31	0.30	0.14	**0.44**	**0.000**
CCF	4.17	22.0	10.7	8.17	5.27	**0.49**	**0.000**
CCS	109	3197	632	454	614	**0.97**	**0.000**
CCC	139	2959	1075	584	907	**0.84**	**0.000**
TSA	0.05	0.47	0.23	0.23	0.10	**0.42**	**0.000**
VN	13.2	44.5	27.3	28.2	6.53	0.24	**0.000**
XVA	0.01	0.07	0.03	0.03	0.01	**0.44**	**0.000**

Twenty-three of 42 Traits with coefficients of variation (CVs) ≥ 0.25 appear in bold type. Probability values were based on a GLM multivariate analysis of 53 alfalfa genotypes and appear in bold if < 0.05

### Variation in local traits

**Fig. 3:**
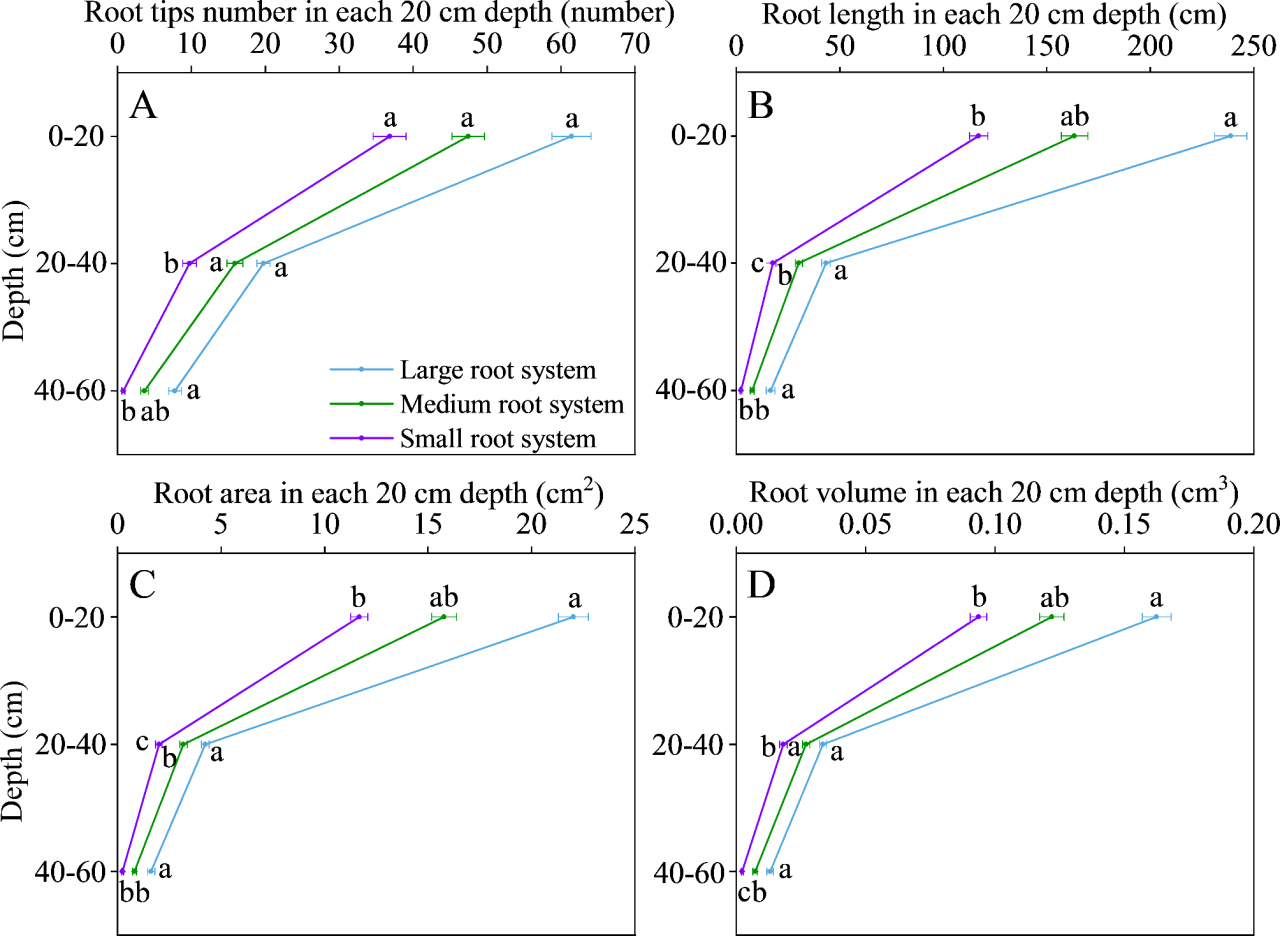
Root tips number **(A)**, root length **(B)**, root area **(C)** and root volume **(D)** distribution in 20 cm increments for three groups of alfalfa genotypes presented by root system size. Different letters indicate significant differences among the three root system size groups (*P* < 0.05)

The local root traits had a larger variation than the global root traits (Table 1). The CVs of all local root traits were greater than 0.25, except for root length in diameter-thick (diameter class ≥ 0.25 mm), which was 0.23. There were significant differences among the tested genotypes in root length in diameter-thin, root tips number-60, root length-60, root area-60 and root volume-60 (*P* < 0.05). The average root diameter of all genotypes was 0.33 mm. The average root length in diameter-thin (diameter class < 0.25 mm) and root length in diameter-thick of all genotypes accounted for 35.7% and 64.3% of the total root length, respectively. On average, the distribution of root tips number, root length, root area and root volume decreased with the increasing soil depth. About 79.0%, 16.0% and 5.00% of the root length across all genotypes were distributed in the 0–20 cm, 20–40 cm and below the 40 cm soil layer, respectively. Genotypes with large root systems had the maximum root tips number, root length, root area and root volume followed by medium and small root systems in each soil layer (Fig. 3). The differences in root distribution among genotypes with different root system sizes were mainly reflected in the soil layer below 20 cm.

### Variation in anatomical traits

**Fig. 4:**
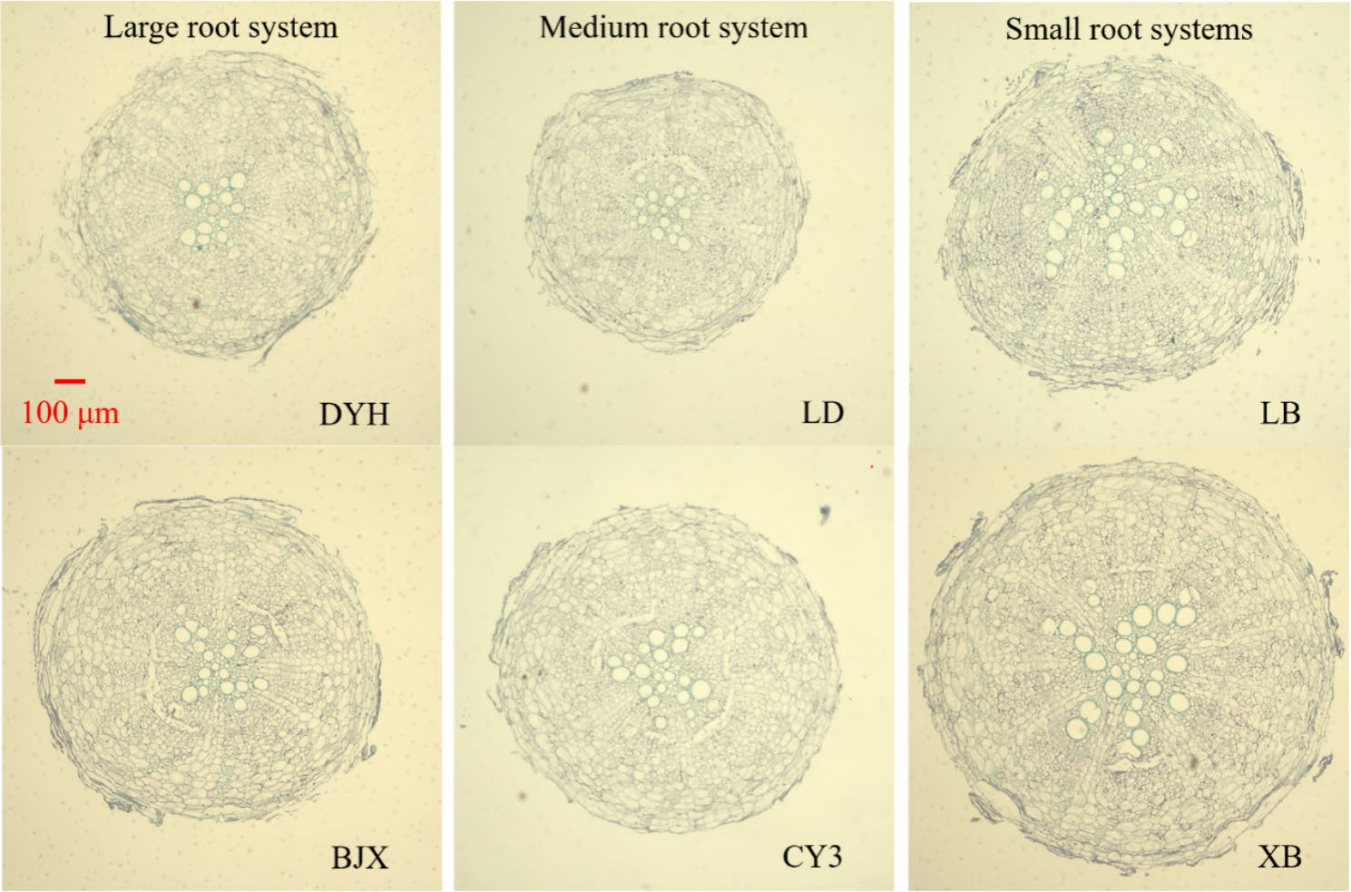
Variation in anatomical features of large root systems (genotypes DYH and BJX), medium root systems (genotypes LD and CY3) and small root systems (genotypes LB and XB). Bar = 100 μm

**Fig. 5:**
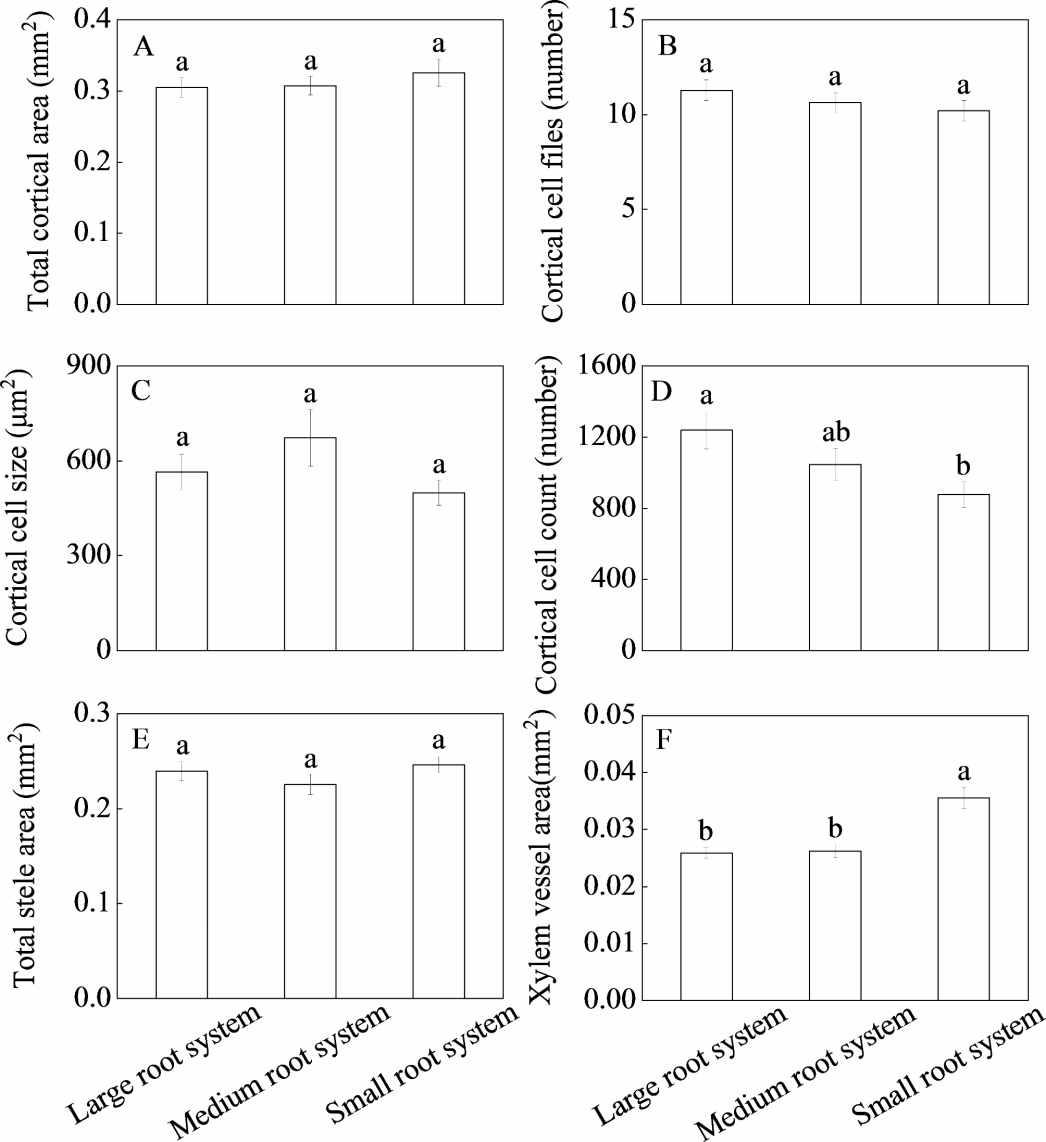
Six root anatomical traits with CVs ≥ 0.25 for three groups of alfalfa genotypes presented by root system size. Different letters indicate significant differences among the three root system size groups (*P* < 0.05)

Except for vessel number, the CVs of other anatomical traits were all higher than 0.25 (Table 1). A significant difference was detected in all tested root anatomical traits across genotypes (*P* < 0.001). Root anatomical features of genotypes DYH and BJX (large root systems), LD and CY3 (medium root systems), and LB and XB (small root systems) were shown in Fig. 4. Cortical cell count of the large root systems was higher than that of the small root systems (Fig. 5). But xylem vessel area of the small root systems was higher than that of the medium and large root systems.

### Correlation among traits

Among 42 measured traits, 4 global traits, 13 local traits and 6 anatomical traits with larger coefficients of variation (CVs ≥ 0.25, Table 1) were used for Pearson’s correlation analysis. Nitrogen uptake, and phosphorus uptake both showed a positive correlation with total cortical area, total stele area and xylem vessel area (Table S1). Total root length and root tips number exhibited a positive correlation and these two traits were positively correlated with all local root traits (*P* < 0.01). Most local traits had significant correlations with each other (*P* < 0.05), such as root length in diameter-thin and root tips number-40 were positively correlated with all local traits (*P* < 0.01).

Root tips number-20 was positively correlated with total stele area, and negatively correlated with total cortical area, cortical cell files and cortical cell count (*P* < 0.05). Root length-40 and root area-40 were both positively correlated with cortical cell files and cortical cell count (*P* < 0.05). Root volume-40 was positively correlated with cortical cell count (*P* < 0.05). The rest of the anatomical traits showed no significant correlation with global and local traits. But most anatomical traits were correlated with each other (*P* < 0.01).

**Fig. 6:**
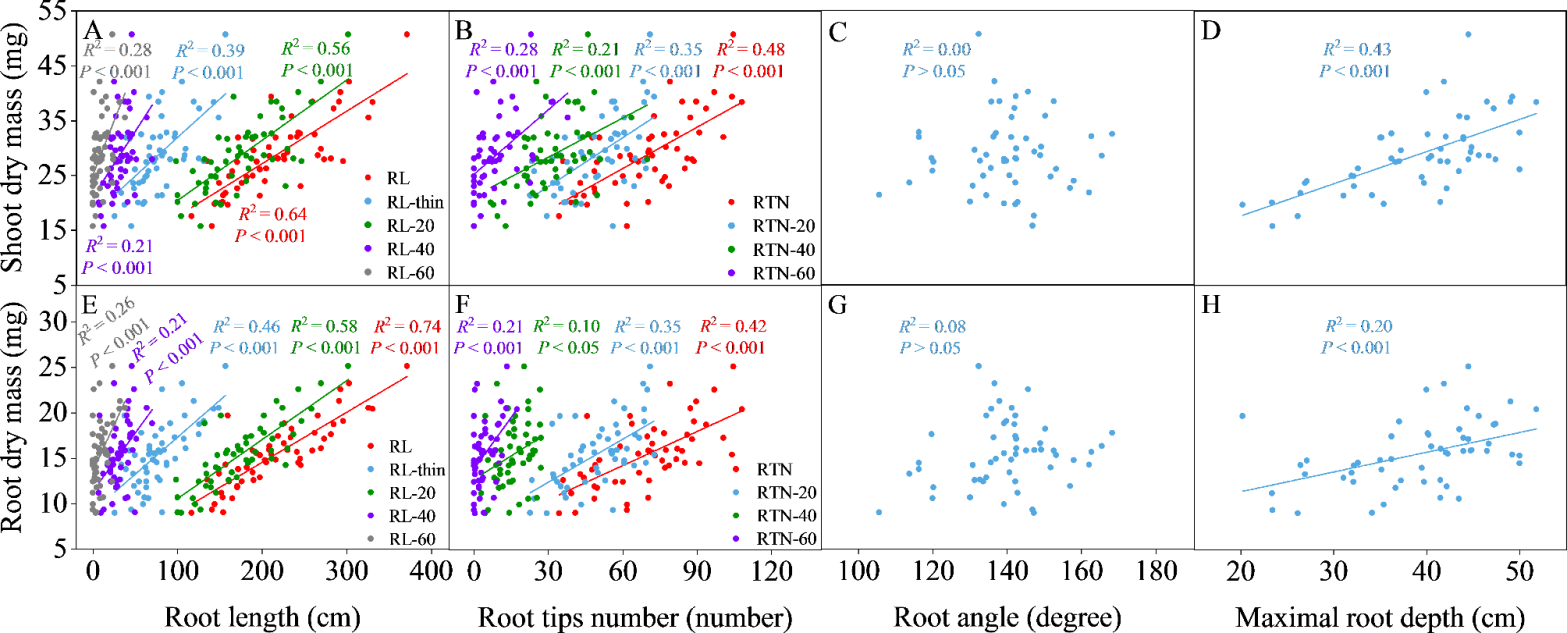
Correlation between **(A)** root length and shoot dry mass, **(B)** root tips number and shoot dry mass, **(C)** root angle and shoot dry mass, **(D)** maximal root depth and shoot dry mass, **(E)** root length and root dry mass, **(F)** root tips number and root dry mass, **(G)** root angle and root dry mass, **(H)** maximal root depth and root dry mass

**Fig. 7:**
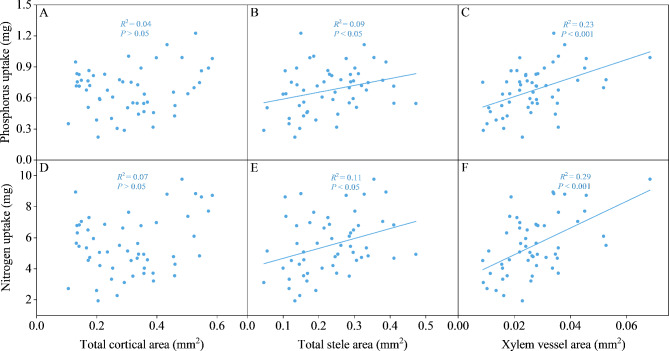
Correlation between **(A)** total cortical area and phosphorus uptake, **(B)** total stele area and phosphorus uptake, **(C)** xylem vessel area and phosphorus uptake, **(D)** total cortical area and nitrogen uptake, **(E)** total stele area and nitrogen uptake, **(F)** xylem vessel area and nitrogen uptake

Moreover, total root length, root length in diameter-thin, root length in each section, root tips number, root tips number in each section and maximal root depth were all positively correlated with shoot dry mass and root dry mass (*P* < 0.05; Fig. 6). Total stele area and xylem vessel area were both positively correlated with phosphorus uptake and nitrogen uptake (*P* < 0.05; Fig. 7).

### Determination of trait variation

Principal component analysis (PCA) was performed for the 23 selected traits with CVs ≥ 0.25 (Table 2). Six principal components (PCs) were identified with eigenvalues > 1, capturing 88.0% of the total variation in these traits across the tested genotypes. PC1 and PC2 represented 52.9% of the variability and consisted of all root morphological traits. PC3 represented 12.9% of the variability and consisted of all the cortical traits, such as total cortical area, cortical cell files, cortical cell size and cortical cell count. PC4 accounted for 10.7% of the variability and consisted of nitrogen uptake, phosphorus uptake, total stele area and xylem vessel area.

**Fig. 8:**
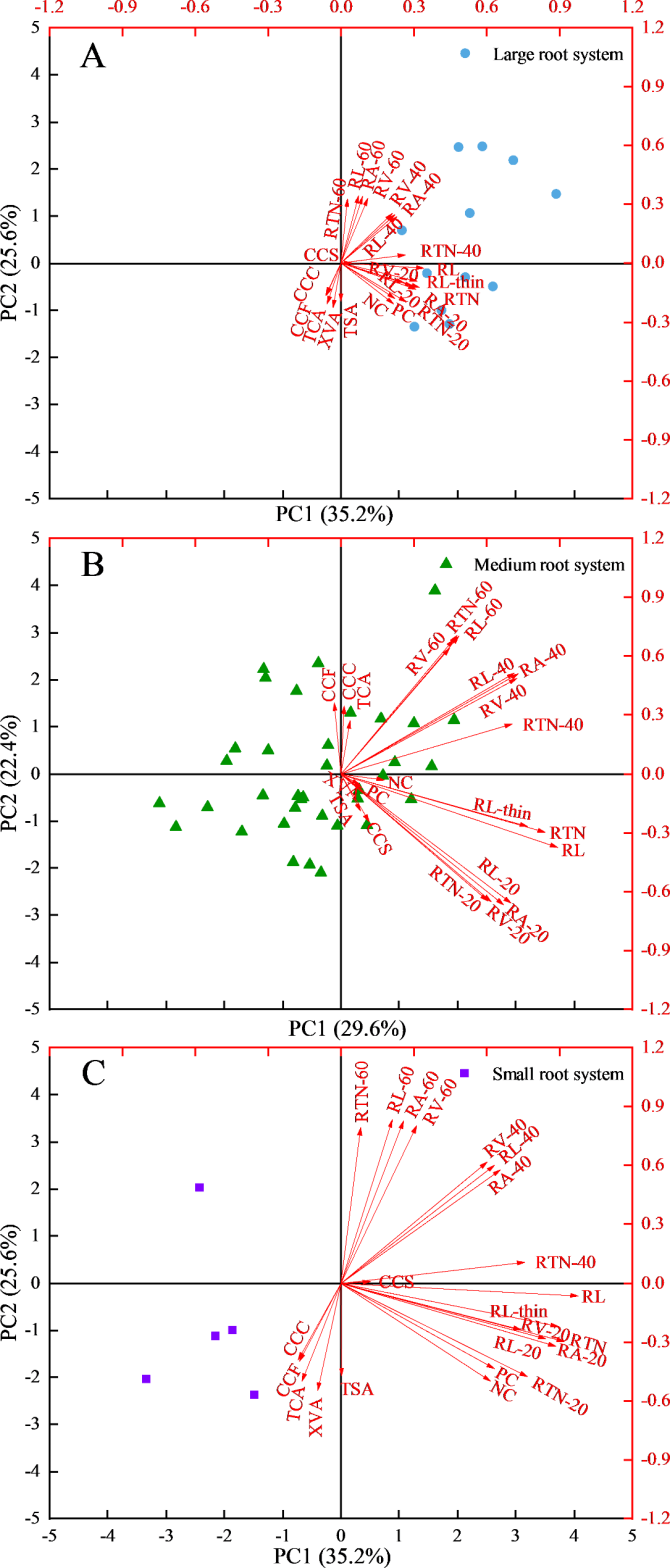
Principal component analysis of 23 selected traits with CVs ≥ 0.25 for three groups of alfalfa genotypes presented by root system size

Besides, principal component analysis was also performed for genotypes with large root systems, medium root systems and small root systems, respectively. And 23 traits with CVs ≥ 0.25 in PC1 and PC2 were shown in Fig. 8. Genotypes with three types of root system size showed a clear separation. Except for total cortical area, cortical cell files, cortical cell count and xylem vessel area, all other traits had positive contributions to large and small root system size. Except for cortical cell files, all other traits had positive contributions to medium root system size. Among the 23 selected traits, total root length, root tips number, root length in diameter-thin, root length-20, root length-60, root area-60, root volume-60 and root tips number-60 contributed the most to the size of the large root system. Total root length, root tips number, root length-60 and root area-60 contributed the most to the size of medium and small root systems. Additionally, root volume-60 contributed greatly to the size of the small root system.

**Table 2:** Principal component (PC) analysis of 23 selected traits with CVs ≥ 0.25 and the proportion of variation in each principal component

Trait	PC1	PC2	PC3	PC4	PC5	PC6
NU	0.06	-0.06	0.46	**0.72**	0.19	-0.42
PU	-0.04	-0.09	0.47	**0.70**	0.13	-0.47
RL	**0.91**	-0.33	0.09	-0.09	0.08	-0.05
RTN	**0.83**	-0.37	0.06	-0.01	0.01	0.13
RL-thin	**0.82**	-0.19	0.12	-0.03	0.09	-0.12
RTN-20	0.59	**-0.68**	0.07	-0.09	0.12	0.09
RTN-40	**0.64**	0.17	0.19	0.14	-0.48	0.14
RTN-60	**0.57**	0.56	-0.32	0.07	0.37	0.03
RL-20	**0.74**	-0.61	0.14	-0.16	0.12	-0.05
RL-40	**0.74**	0.45	0.10	0.16	-0.37	-0.02
RL-60	0.56	**0.63**	-0.35	0.06	0.36	-0.01
RA-20	**0.71**	-0.63	0.11	-0.18	0.13	-0.02
RA-40	**0.74**	0.45	0.09	0.16	-0.44	-0.01
RA-60	0.58	**0.64**	-0.35	0.07	0.35	0.00
RV-20	**0.65**	-0.63	0.08	-0.20	0.12	0.02
RV-40	**0.70**	0.43	0.08	0.16	-0.48	0.01
RV-60	0.57	**0.63**	-0.34	0.08	0.32	0.01
TCA	-0.05	0.36	**0.78**	-0.07	0.19	0.35
CCF	0.00	0.48	**0.76**	-0.36	0.15	0.07
CCS	0.08	-0.28	**-0.32**	0.29	-0.25	0.14
CCC	0.03	0.45	**0.79**	-0.31	0.11	0.07
TSA	-0.02	-0.31	-0.02	**0.64**	0.13	0.44
XVA	-0.10	-0.16	0.18	**0.71**	0.18	0.50
Eigenvalue	7.34	4.82	2.96	2.47	1.59	1.05
Contributive ratio (%)	31.9	20.9	12.9	10.7	6.91	4.58
Cumulative contributive ratio (%)	31.9	52.9	65.7	76.5	83.4	88.0

For each trait, the largest absolutely variable loading score crossing the six components appears in bold. Principal components with eigenvalues > 1 are presented and considered significant

### Genotype distribution based on root trait variation

Based on the composite score of PCA, 12 genotypes with large root systems, 36 genotypes with medium root systems, and 5 genotypes with small root systems were ranked from the least to the most, respectively (Fig. S1). The average composite score of large root system was significantly higher than that of the medium root system and small root system.

The dendrogram of agglomerative hierarchical clustering (AHC) separated the 53 genotypes into five major groups at a rescaled distance of 15 using the average linkage method with squared Euclidean distance as the interval measurement on the same set of 23 traits with CVs ≥ 0.25 (Fig. S2). This revealed variation in the degree of homogeneity among genotypes tested. Groups 1 to 5 had 27, 11, 6, 5 and 4 genotypes, respectively. Genotypes with large root systems were distributed in groups 1, 4 and 5, genotypes with medium root systems were distributed in groups 1, 2, 3 and 4, and genotypes with small root systems distributed in groups 1, 2 and 3. This suggests that genotypes with the same root system were not always clustered in the same or closer groups.

Fifty-three genotypes were arranged in 5 groups determined by AHC and ranked according to the composite score in each group (Fig. S3). Ten genotypes with the highest composite score in each group presented by root system size (genotypes BJX, JG, LB, G3, B218, MTW, MF, Z2, AH and PL) were selected for further study. The root traits of 10 alfalfa genotypes selected were significantly different, and the ranking of 23 traits (CVs ≥ 0.25) of each genotype was significantly different among 53 genotypes (Table S2). For example, BJX and MF had obvious differences in the ranking of all traits. JG and LB had a similar total cortical area (ranked 26th and 30th, respectively), cortical cell size (ranked 8th and 6th, respectively) and xylem vessel area (ranked 8th and 2th, respectively), but there were significant differences in other root traits between them.

## Discussion

### Variations among root traits and its implications for alfalfa breeding

Selection for root traits as a vital strategy of breeding has received more and more attention [5, 29]. Among the 36 root traits we measured, 2 global root traits, 13 local root traits and 6 root anatomical traits exhibited larger variation among tested genotypes (CVs ≥ 0.25). The magnitude of variation in local root traits and root anatomical traits was higher than that of global root traits, which was consistent with the research results in maize roots [30] and wheat roots [31]. Variations in root anatomical traits can affect the acquisition efficiency of water and nutrients [32–34]. Therefore, we should pay more attention to the differences in local morphological traits and anatomical traits in the root research of alfalfa.

RSA and root anatomy at the seedling stage are highly correlated with crop yield [28, 35]. Higher number of adventitious roots and taproot length in common bean seedlings contributed to water and nutrient uptake, leading to a significant positive correlation with yield [36]. Increasing the number and area of vessels in maize seedlings improved root hydraulic conductivity and yield under drought condition [35]. In the present study, significant differences were detected in root angle, maximal root depth, root width, branch intensity, root length in diameter-thin, root tips number-60, root length-60, root area-60, root volume-60 and all the tested root anatomical traits among the tested genotypes. The above traits with significant differences may be the key factors influencing root absorptive capacity and plant growth at the seedling stage.

Plant breeding based on root traits has lagged behind that based on aboveground traits because the roots of plants are difficult to measure [37]. Besides, the characteristics of alfalfa such as heterogeneous pollination, polyploid inheritance and self-incompatibility have hindered the molecular breeding of alfalfa [38]. Through comprehensive multiple analysis, 10 genotypes with different root traits were selected in this study. Some studies have shown the special advantages of the individual alfalfa genotype selected here. The genotype B218 with a small root system is sensitive to autotoxicity [39]. Autotoxicity can inhibit root growth during the seedling stage, which may lead to differences in root size [40]. Genotype AH with a medium root system had strong drought tolerance by increasing root branching to absorb more water [41]. These selected genotypes could be used for studying their adaptive mechanism under abiotic stresses and provide a reference for the selection of candidate parents.

### Contribution of individual root traits to resource absorption

The 44 root-related traits measured in this study reflected alfalfa root growth (such as total root length and root tips number), root distribution (such as maximal root depth, root width and root angle) and root internal structure (such as total cortical area, total stele area and vessel number). Root system size (root length and root mass) is one of the important factors for plants to exploit soil resources [8, 42]. Large root systems can uptake more nitrogen and water contributing to plant growth and competitiveness than small root systems in early growth stages [43]. While, under high planting density, oversized root systems may intensify intraspecific competition and result in yield reduction [44]. Root biomass and root length of modern wheat varieties are smaller than those of past varieties, but the yield is increased [45]. Therefore, a large root system is not always a beneficial trait. Under different soil conditions, the optimal root system is different [21]. In this study, there was no significant difference in nitrogen and phosphorus uptake among genotypes with three root system sizes. Therefore, a larger root system did not guarantee greater nutrient absorption in alfalfa seedlings. Nutrient uptake is also influenced by arbuscular mycorrhizal and root exudations [46, 47].

The distribution of the root system in the soil is closely related to its absorption strategy [5]. Deep-rooting improves drought tolerance, nitrogen accumulation and harvest index of rice during grain filling [48]. Our data showed that maximal root depth was positively correlated with root biomass and shoot biomass, suggesting that deep-rooting can help alfalfa to improve its growth at the seedling stage. Steeper root growth angles can expedite the development of deeper roots, which aids in the efficient utilization of resources located in deeper soil layers, especially nitrogen and water [4]. While shallow root angles play a crucial role in foraging capacity of plants in the topsoil layer, especially in the absorption of phosphorus [49, 50]. Genotypes with different root maximal depth and root angle can be used for further studies about drought and nutrient stress. Root length and number of root tips are important parameters for evaluating water and nutrient uptake capacity [51, 52]. In this study, root length and number of root tips in different soil layers were positively correlated with root and shoot biomass. Longer root length increases the root-soil contact area, facilitating the uptake of nitrogen, phosphorus, and kalium in wheat [10]. More root tips help to capture nitrogen and water in citrus rootstocks [53]. Therefore, a higher number of root tips and root length of the genotypes with large root systems contribute more to shoot dry mass accumulation than the genotypes with small root systems. But root length and number of root tips in different soil layers did not exhibit significant correlation with nitrogen and phosphorus uptake, probably because nitrogen and phosphorus uptake are also influenced by factors such as root anatomical characteristics [4]. Meanwhile, the experiment was conducted at the seedling stage and the rhizobox limited the lateral growth of the root system. The relationship between these root traits and nutrient uptake needs to be verified in the field.

The performance of root anatomy is important for the acquisition and transportation of nutrients and water within the plant, and the costs and benefits associated with root growth [54]. In this study, all the anatomical traits measured exhibited evident variations across the tested genotypes. Variations in these traits have notable effects on the acquisition efficiency of nutrient and water [5]. Larger vessel diameter contributes to higher root axial hydraulic conductance in peach rootstocks [55]. The larger stele diameter can enhance the capacity of crops to penetrate the soil and improve phosphorus uptake to better adapt to drought stress [19]. In this study, total stele area and xylem vessel area were both positively correlated with nitrogen and phosphorus uptake, indicating that larger total stele area and xylem vessel area could improve nitrogen and phosphorus uptake by reducing axial transport resistance in alfalfa at the seedling stage. Genotypes with three root system size groups showed differences in xylem vessel area reflecting the differences in water transportation. Alfalfa genotypes with small root systems had larger xylem vessel area, enabling them to improve axial transport of water better adapt to environmental stresses at the seedling stage.

RSA and anatomy are closely linked because root anatomy has important implications for root architecture and metabolic cost [56]. Root metabolic cost affects RSA directly by altering the number and length of roots, and indirectly by affecting access resources from soil [4]. In this study, only root tips number-20, root length-40, root area-40 and root volume-40 correlated with anatomic traits. In addition, anatomic and morphologic traits showed separation in principal component analysis. An integrated understanding of both morphologic and anatomic characters is necessary to better realize the adaptive mechanisms of the alfalfa root system in future studies.

## Conclusions

Larger variations were observed in 21 root morphological and anatomical characteristics among the 53 tested alfalfa genotypes. The extent of variability was more pronounced for local root traits and root anatomical features when compared to global root traits. Alfalfa genotypes with distinct root system sizes exhibited differences in root distribution and xylem vessel area, which could potentially influence the absorption and transportation of resources in alfalfa seedling roots. Total root length, root length in diameter thin, root tips number, root length and root tips number in different soil layers, as well as maximal root depth showed positive correlations with shoot and root dry mass. Additionally, total stele area and xylem vessel area displayed strong correlations with nitrogen and phosphorus uptake. These valuable root traits, associated with biomass and nutrient absorption, could be integrated into marker-assisted selection strategies for breeding improved alfalfa genotypes with enhanced resource absorption and overall plant growth. In future studies, the integration of root morphology, anatomy and molecular biology will be essential to advance our understanding of the root adaptation mechanism in alfalfa and to facilitate the development of new alfalfa cultivars with improved resource uptake and adaptability to various environments.

### Methods

#### Plant material

A collection of 53 genotypes of alfalfa (*Medicago sativa* L.) was used in this study, including 12 genotypes from China (9 breeding lines and 3 native cultivars) and 41 genotypes from other countries (Table S1). These genotypes were widely grown in China, Canada, Australia, America and Europe, and could effectively represent the genetic diversity of alfalfa. The seeds of the 41 introduced genotypes were obtained from Barenbrug International Grass Industry Co., Ltd (Tianjin, China). The seeds of the 12 Chinese genotypes were obtained from Inner Mongolia Agricultural University, Gansu Grassland Ecology and Xinjiang Agricultural University.

### Growth conditions

**Fig. 9:**
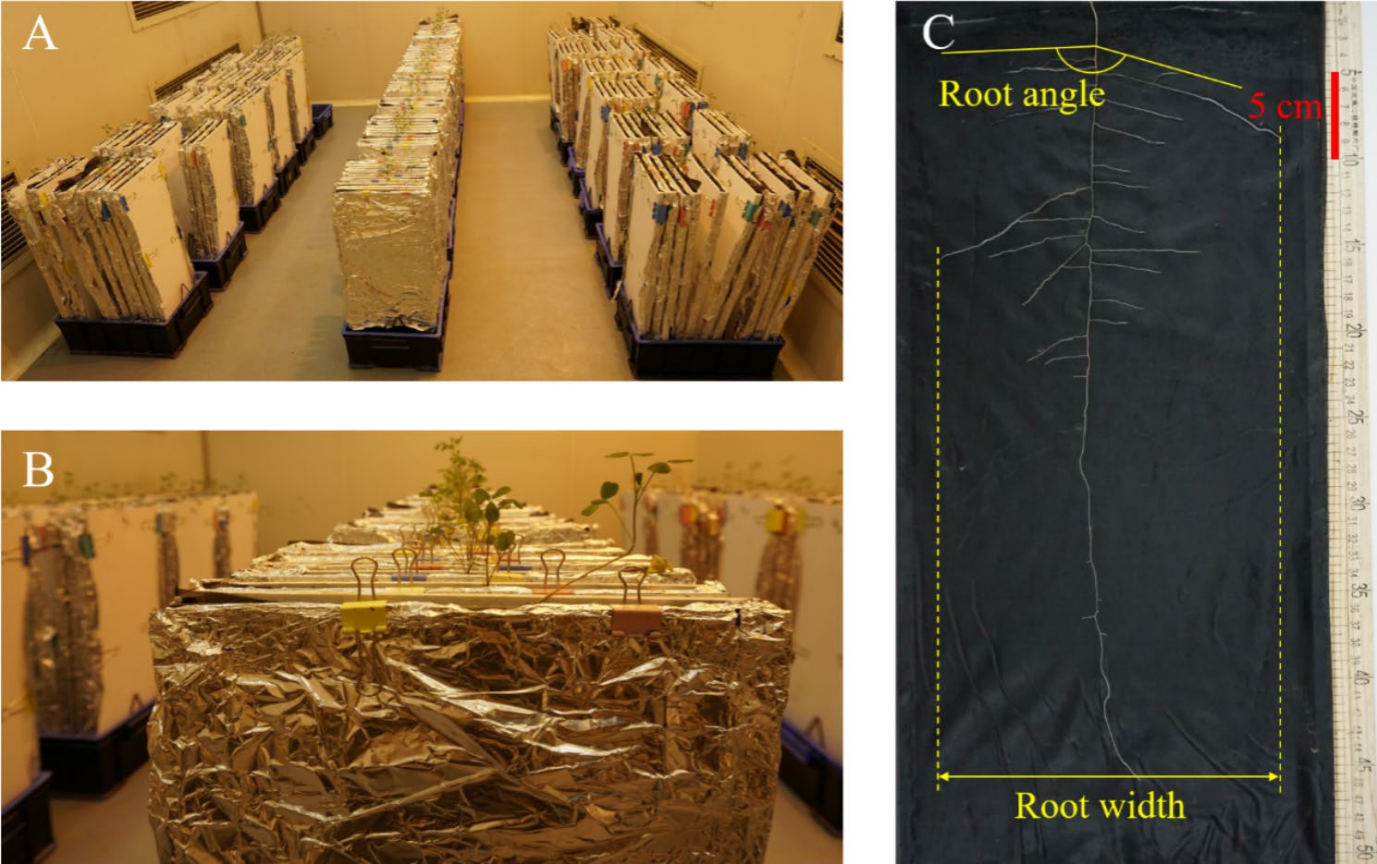
Alfalfa plants grown in rhizoboxes, 45 days after sowing **(A, B)** and an example of root system of genotype B218 **(C)**. Measurements of root angle and root width were indicated. Bar = 5 cm

Rhizoboxes (30 × 2 × 60 cm, length × internal width × depth) were used in this experiment (Fig. 9a and b). Each rhizobox contained a transparent polycarbonate (PC) board on one side and a white polyvinyl chloride (PVC) foam board on the other side. A black nylon cloth of the same size was attached to the PC board. The bottom of the rhizobox was wrapped with gauze to prevent quartz sand from leaking out. We used 41 mm long tail clips to fix the nylon cloth, bottom, PVC and PC board together. The PC board was covered with tin foil to avoid light exposure to the roots. After the rhizoboxes were assembled, 4 kg of washed quartz sand with a diameter of about 2 mm was filled between the black nylon cloth and the PVC board in each rhizobox. The rhizoboxes were placed vertically by tripod in a plastic box filled with 6 L of Hoagland nutrient solution.

The healthy alfalfa seeds of uniform size were disinfected and rinsed [57], and then sown about 2 cm from the soil surface in the rhizoboxes. The seeds were sown between black nylon cloth and PC board. Each rhizobox retained one plant, and each replicate retained plants with similar sizes. Three biological replicates were established for each genotype, and they were planted in three different rhizoboxes. Ten rhizoboxes were placed in each plastic box, and a total of 159 rhizoboxes were placed in 16 plastic boxes. Six plastic boxes were arranged in a row for a total of 3 columns. Rhizoboxes in the same column were moved longitudinally once a week, and the positions of different columns were exchanged to reduce the influence of environmental factors on plant growth.

The experiment was conducted in a climate chamber (the day/night temperature was 25℃/20℃, the humidity was 65%, the lighting time was from 7:00 to 19:00, and the lighting intensity was 800 µmol m^− 2^ s^− 1^) at Northwest A&F University, Yangling (34°16′ N, 108°4″ E) in June 17 to July 31, 2020. Rhizoboxes were rinsed with ultrapure water every 7 days to prevent salt accumulation in the sand culture and then freshly nutrient solution was poured into the plastic box. The Hoagland nutrient solution was consisted of (mmol L^− 1^) N (4.00), K (1.50), Ca (1.00), P (0.50), S (0.50), Mg (0.50), Cu (3.96 × 10^− 4^), Zn (9.56 × 10^− 4^), Mn (1.14 × 10^− 2^), Cl (2.28 × 10^− 2^), B (5.78 × 10^− 2^), Mo (2.62 × 10^− 4^) and Fe (6.72 × 10^− 2^). The pH of the nutrient solution was 5.8.

### Sampling and measurements

Plants were harvested after 45 days when the first plant reached the bottom of the rhizobox. Simultaneously, approximately one-fifth of the plants had their root systems reaching the bottom of the rhizoboxes. At harvest, the number of trefoil leaves and shoot height were measured. Then, shoots were cut from the roots and dried in an air-forced oven at 75 °C for 72 h to determine shoot dry mass.

Root angle was measured with a digital angle ruler [29, 30], maximal root depth and root width were measured with a ruler (Fig. 9c). And the number of root tips per 20 cm soil layer was counted manually. Root systems were photographed with a camera at a fixed height (Fig. 9c). The above root measurements and photography were taken while the root systems were still in the rhizoboxes. After photographing, root subsamples were collected by cutting the root system into 20-cm sections starting from the base. Root samples were cleaned with deionized water and separated without overlapping in a special root tray. Root subsamples were scanned in greyscale at 300 dpi using a desktop scanner (Epson Perfection, V800, Long Beach, CA, USA). The images were analyzed using WinRHIZO Pro (v2009, Regent Instruments, Montreal, QC, Canada) to obtain root morphological characteristics including root length, root area, root volume, average root diameter and root length in two diameter classes [30]. It is recognized that root diameter less than 0.2 mm are fine roots in herbaceous plants [58]. Here, roots with diameter < 0.25 mm were classified as thin roots, while roots with diameter ≥ 0.25 mm were classified as thick roots.

After scanning, a 1 cm long segment of root was cut at 5 cm from the taproot tip, then preserved in formaldehyde-acetic acid-ethanol fixative (75% ethanol, glacial acetic acid, 40% formaldehyde) and stored at 4 °C until further analysis [19, 23]. The segments were embedded in paraffin individually after dehydration by immersion in a sequence of alcohol solutions. The roots were then cut into sections with a thickness of 5 μm using LEICA automatic microtome. The slices were fully baked and stained with toluidine blue. Slices were viewed under MoticBA410 optical microscope at 4 × magnification with an additional 0.65 × adapter, then photographed and saved using Motic Images Advanced 3.2 software. ROOTSCAN 2.4 software was used to analyze the pictures to obtain the corresponding root anatomical traits data including total cortical area, cortical cell files, cortical cell size, cortical cell count, total stele area, vessel number and xylem vessel area [54]. Area measurements were in mm^2^ and calibrated from pixels using an image of a 1-mm micrometer taken at the same magnification as the analyzed images (1 linear mm = 1215 pixels).

Root subsamples from the same plant were combined into one root sample and dried in an air-forced oven at 75 °C for 72 h to obtain root dry mass. Dry shoot and root samples were ground by a high-speed grinder MM400 (Retsch, Germany) and then digested with concentrated H_2_SO_4_-H_2_O_2_. The total N concentration of root and shoot was determined by the Kjeldahl method and the total P concentration was determined by a molybdenum-antimony colorimetric method [59].

The following traits were calculated from the measured data: N/P uptake (NU/PU) = shoot dry weight × N/P concentration in shoot + root dry weight × N/P concentration in root [47, 60]; specific root length (SRL) = total root length divided by root dry mass; specific root area (SRA) = total root area divided by root dry mass; root to shoot mass ratio (RSM) = root dry mass divided by shoot dry mass; root tissue density (RTD) = root dry mass divided by total root volume; root length intensity (RLI) = total root length divided by root depth [8]; branch intensity (BI) = root tip number divided by total root length [61].

The 42 traits were divided into three general categories: 21 global traits, 14 local traits and 7 anatomical traits (Table 3). Global traits refer to the whole root system, whole shoots and N/P uptake, and local traits refer to roots in different depths and diameter classes [29, 30, 61].

**Table 3:** Description of 42 measured traits (21 global traits, 14 local traits and 7 root anatomical traits) of 53 alfalfa genotypes

Traits	Abbreviation	Description	Units
**Global traits**		**Traits at the whole plant level**	
Trefoil number	TN	Number of trefoil leaves per plant	Number
Shoot height	SH	Shoot height (maximal physical height in nature)	cm
Nitrogen uptake	NU	Total nitrogen per plant	mg
Phosphorus uptake	PU	Total phosphorus per plant	mg
Shoot dry mass	SDM	Total shoot dry mass per plant	mg
Root dry mass	RDM	Total root dry mass per plant	mg
Total dry mass	TDM	Total dry mass per plant	mg
Root angle	RA	The maximal growth angle between two outer lateral roots	Degree
Maximal root depth	MRD	The maximal vertical depth of root	cm
Root width	RW	The maximal extent of the root system in horizontal direction	cm
Root diameter	RD	Average root diameter per plant	mm
Total root length	RL	Total length of all roots per plant	cm
Total root area	RSA	Total surface area of all roots per plant	cm^2^
Total root volume	RV	Total volume of all roots per plant	cm^3^
Root tips number	RTN	Number of root tips per plant	Number
Specific root length	SRL	Total root length divided by root dry mass	cm mg^− 1^
Specific root area	SRA	Total root area divided by root dry mass	cm^2^ mg^− 1^
Root to shoot mass ratio	RSM	Total root dry mass divided by shoot dry mass	
Root tissue density	RTD	Root dry mass per unit root volume	mg cm^− 3^
Root length intensity	RLI	Total root length per unit root depth	cm cm^− 1^
Branch intensity	BI	Root tip number per unit total root length	root cm^− 1^
**Local traits**		**Traits at local level including ratios**	
Root length in diameter-thin	RL-thin	Root length of “thin roots” (in diameter class < 0.25 mm)	cm
Root length in diameter-thick	RL-thick	Root length of “thick roots” (in diameter class ≥ 0.25 mm)	cm
Root tips number-20	RTN-20	Number of root tips per plant in 0–20 cm layer	Number
Root tips number-40	RTN-40	Number of root tips per plant in 20–40 cm layer	Number
Root tips number-60	RTN-60	Number of root tips per plant in 40–60 cm layer	Number
Root length-20	RL-20	Root length in 0–20 cm layer	cm
Root length-40	RL-40	Root length in 20–40 cm layer	cm
Root length-60	RL-60	Root length in 40–60 cm layer	cm
Root area-20	RSA-20	Root surface area in 0–20 cm layer	cm^2^
Root area-40	RSA-40	Root surface area in 20–40 cm layer	cm^2^
Root area-60	RSA-60	Root surface area in 40–60 cm layer	cm^2^
Root volume-20	RV-20	Root volume in 0–20 cm layer	cm^3^
Root volume-40	RV-40	Root volume in 20–40 cm layer	cm^3^
Root volume-60	RV-60	Root volume in 40–60 cm layer	cm^3^
**Anatomical structure traits**		**Primary root anatomical structure traits**	
Total cortical area	TCA	Total area of cortical region	mm^2^
Cortical cell files	CCF	Total number of radial cortical growth rings	Number
Cortical cell size	CCS	Average size of cortical cells	µm
Cortical cell count	CCC	Total number of cortical cells	Number
Total stele area	TSA	Total area of stele region	mm^2^
Vessel number	VN	Total number of xylem vessels in stele region	Number
Xylem vessel area	XVA	Total cross-sectional area of all metaxylem vessels	mm^2^

### Statistical analysis

Based on total root length per plant, genotypes were divided into three groups with small, medium or large root systems [8]. The medium root-sized group interval was defined as the median value of RL (208.48 cm plant^− 1^) ± standard deviation (57.04 cm plant^− 1^) and the upper and lower boundaries of the medium interval were constructed by adding to or subtracting from the median point.

One-way ANOVA and Duncan were conducted using SPSS Statistics 21.0 (IBM Corp, Armonk, NY, USA) for significant differences among the tested genotypes for each trait (*P* < 0.05). Traits with coefficients of variation (CV, standard deviation divided by mean) ≥ 0.25 were selected for Pearson correlation analysis, hierarchical cluster analysis and principal component analysis. When *P* ≤ 0.05, correlations were considered statistically significant. Hierarchical cluster analysis was used to determine the homogeneous groups among genotypes using the average linkage method. Principal component analysis was used to identify the determinants of variability in RSA and anatomical traits across genotypes [30, 62]. The composite score based on principal component analysis was used to obtain a general ranking order for each genotype. The composite score was calculated as follows [30]:

$${X_j} = {a_{1j}}{R_{1j}} + {a_{2j}}{R_{2j}} + \ldots + {a_{ij}}{R_{ij}}i,{\text{ }}j = 1,{\text{ }}2,{\text{ }}3,{\text{ }} \ldots ,n$$

$${\text{D}} = \sum\limits_{j = 1}^n {\left( {{{\text{X}}_j} \times {{\text{W}}_j}} \right)} \,\,\,\,\,\,\,\,j = 1,2,3,....,n$$

In the formula, X_j_ represents the value of the j^th^ comprehensive index in the component matrix, a_ij_ represents the eigenvector corresponding to the eigenvalues of each single index, R_ij_ is the standardized value of each single index, and W_j_ represents the importance of the j^th^ comprehensive index among all the comprehensive indexes. And D represents the composite score of each genotype. The a_ij_, R_ij_ and W_j_ were acquired from principal component analysis. Figures were plotted using Origin 2018 (OriginLab, Northampton, Massachusetts, USA). The value of each trait was the mean of 3 biological replicates, and was expressed as mean ± standard error in the graphs.

### Electronic supplementary material

Below is the link to the electronic supplementary material.

Supplementary Material 1

## Data Availability

Data will be made available on request.
