# Supplementary material for: Exploring root system architecture and anatomical variability in alfalfa (Medicago sativa L.) seedlings
Source: BMC Plant Biol. 2023 Sep 25;23:449. doi: 10.1186/s12870-023-04469-4 (PMC10519072; doi:10.1186/s12870-023-04469-4)
Supplement: Supplementary file 1 — Supplementary Material 1 [file 12870_2023_4469_MOESM1_ESM.docx]

**Exploring root** **system** **architecture** **and anatomical variability in alfalfa (*Medicago sativa* L.) seedlings**

Xinya Pan^1^, Pengfei Wang^1^, Xianwei Wei^1^, Jinxin Zhang^1^, Bingcheng Xu^1, 2^, Yinglong Chen^3^, Gehong Wei^4^, Zhi Wang^1, 2, *^

^1^College of Grassland Agriculture, Northwest A&F University, 3 Taicheng Road, 712100 Yangling, China

^2^State Key Laboratory of Soil Erosion and Dryland Farming on the Loess Plateau, Northwest A&F University, 26 Xinong Road, 712100 Yangling, China

^3^The UWA Institute of Agriculture, School of Agriculture and Environment, The University of Western Australia, Perth, WA 6001, Australia

^4^College of Life Sciences, Northwest A&F University, 22 Xinong Road, 712100 Yangling, China

* Correspondence: [wangzhi712@nwafu.edu.cn](mailto:wangzhi712@nwafu.edu.cn)

**Table S1** Pearson’s correlation matrix for 23 traits with CVs ≥ 0.25 in 53 alfalfa genotypes.

|  | P  U | R  L | R  T  N | RL  -thin | RTN  -20 | RTN  -40 | RTN  -60 | RL  -20 | RL  -40 | RL  -60 | RA  -20 | RA  -40 | RA  -60 | RV  -20 | RV  -40 | RV  -60 | T  C  A | C  C  F | C  C  S | C  C  C | T  S  A | X  V  A |
| --- | --- | --- | --- | --- | --- | --- | --- | --- | --- | --- | --- | --- | --- | --- | --- | --- | --- | --- | --- | --- | --- | --- |
| NU | ** |  |  |  |  |  |  |  |  |  |  |  |  |  |  |  | * |  |  |  | ** | ** |
| PU |  |  |  |  |  |  |  |  |  |  |  |  |  |  |  |  | * |  |  |  | ** | ** |
| RL |  |  | ** | ** | ** | ** | ** | ** | ** | ** | ** | ** | ** | ** | ** | ** |  |  |  |  |  |  |
| RTN |  |  |  | ** | ** | ** | ** | ** | ** | ** | ** | ** | ** | ** | ** | ** |  |  |  |  |  |  |
| RL-thin |  |  |  |  | ** | ** | ** | ** | ** | ** | ** | ** | ** | ** | ** | ** |  |  |  |  |  |  |
| RTN-20 |  |  |  |  |  | ** |  | ** |  |  | ** |  |  | ** |  |  | * | * |  | * | * |  |
| RTN-40 |  |  |  |  |  |  | ** | ** | ** | ** | ** | ** | ** | ** | ** | ** |  |  |  |  |  |  |
| RTN-60 |  |  |  |  |  |  |  |  | ** | ** |  | ** | ** |  | ** | ** |  |  |  |  |  |  |
| RL-20 |  |  |  |  |  |  |  |  | ** |  | ** | ** |  | ** | * |  |  |  |  |  |  |  |
| RL-40 |  |  |  |  |  |  |  |  |  | ** | * | ** | ** |  | ** | ** |  | * |  | ** |  |  |
| RL-60 |  |  |  |  |  |  |  |  |  |  |  | ** | ** |  | ** | ** |  |  |  |  |  |  |
| RA-20 |  |  |  |  |  |  |  |  |  |  |  | * |  | ** | * |  |  |  |  |  |  |  |
| RA-40 |  |  |  |  |  |  |  |  |  |  |  |  | ** |  | ** | ** |  | * |  | * |  |  |
| RA-60 |  |  |  |  |  |  |  |  |  |  |  |  |  |  | ** | ** |  |  |  |  |  |  |
| RV-20 |  |  |  |  |  |  |  |  |  |  |  |  |  |  |  |  |  |  |  |  |  |  |
| RV-40 |  |  |  |  |  |  |  |  |  |  |  |  |  |  |  | ** |  |  |  | * |  |  |
| RV-60 |  |  |  |  |  |  |  |  |  |  |  |  |  |  |  |  |  |  |  |  |  |  |
| TCA |  |  |  |  |  |  |  |  |  |  |  |  |  |  |  |  |  | ** | ** | ** |  | ** |
| CCF |  |  |  |  |  |  |  |  |  |  |  |  |  |  |  |  |  |  | ** | ** | ** |  |
| CCS |  |  |  |  |  |  |  |  |  |  |  |  |  |  |  |  |  |  |  | ** |  | ** |
| CCC |  |  |  |  |  |  |  |  |  |  |  |  |  |  |  |  |  |  |  |  | ** |  |
| TSA |  |  |  |  |  |  |  |  |  |  |  |  |  |  |  |  |  |  |  |  |  | ** |

* and ** is significant positive correlation at the 0.05 and 0.01, respectively; * and ** is significant negative correlation at the 0.05 and 0.01, respective

**Fig. S1** The ranking of composite score based on principal component analysis of 53 alfalfa genotypes. Different letters represent significant differences among the three root system size groups (*P* < 0.05)


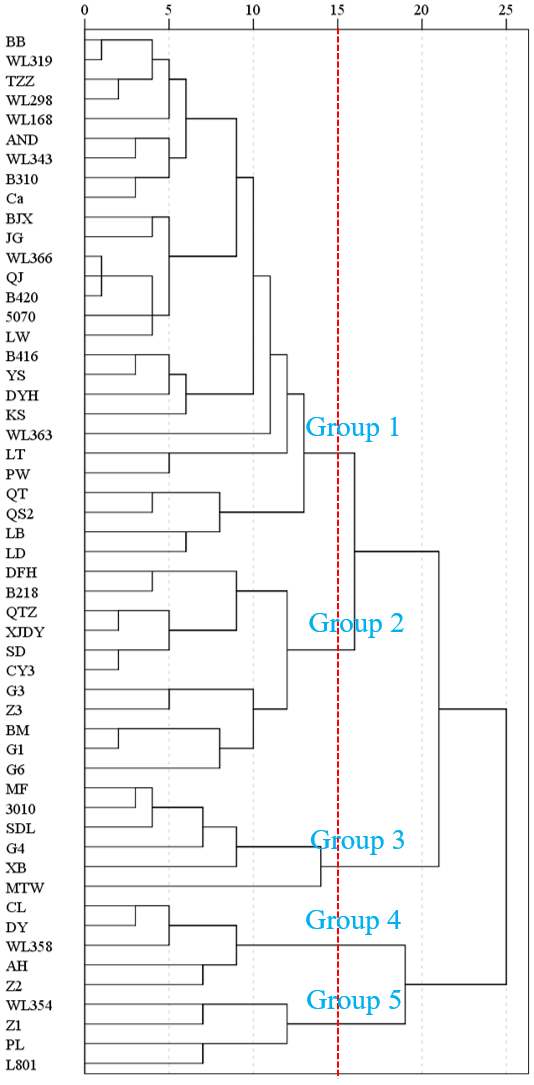


**Fig. S2** Dendrogram of agglomerative hierarchical clustering for 53 alfalfa genotypes.

**Fig. S3** The distribution of 53 alfalfa genotypes in five groups at a middle distance of 15 based on a combination of principal component analysis, hierarchical clustering analysis and composite score.

**Table S2** The ranking of 10 selected genotypes from 53 genotypes for each of 23 selected traits with CVs ≥ 0.25.

| Traits | Large root system | | | Medium root system | | | | Small root system | | |
| --- | --- | --- | --- | --- | --- | --- | --- | --- | --- | --- |
|  | BJX | Z2 | PL | JG | G3 | MTW | AH | LB | B218 | MF |
| **Global traits** |  |  |  |  |  |  |  |  |  |  |
| NC | 17 | 6 | 42 | 8 | 48 | 3 | 9 | 24 | 50 | 5 |
| PC | 19 | 11 | 32 | 3 | 44 | 1 | 31 | 16 | 49 | 6 |
| RL | 8 | 12 | 7 | 17 | 47 | 46 | 22 | 49 | 52 | 50 |
| RTN | 6 | 35 | 8 | 7 | 30 | 50 | 34 | 49 | 52 | 37 |
| **Local traits** |  |  |  |  |  |  |  |  |  |  |
| RL-thin | 5 | 9 | 7 | 16 | 38 | 18 | 14 | 46 | 52 | 39 |
| RTN-20 | 17 | 45 | 19 | 13 | 41 | 53 | 49 | 48 | 52 | 35 |
| RTN-40 | 4 | 23 | 12 | 19 | 5 | 32 | 15 | 41 | 43 | 22 |
| RTN-60 | 9 | 4 | 6 | 7 | 13 | 21 | 8 | 43 | 24 | 50 |
| RL-20 | 10 | 15 | 11 | 22 | 51 | 52 | 45 | 42 | 53 | 48 |
| RL-40 | 11 | 17 | 12 | 13 | 14 | 4 | 1 | 50 | 26 | 40 |
| RL-60 | 10 | 5 | 2 | 8 | 15 | 33 | 4 | 39 | 30 | 50 |
| RA-20 | 15 | 17 | 13 | 23 | 52 | 53 | 46 | 44 | 50 | 49 |
| RA-40 | 12 | 14 | 6 | 9 | 10 | 13 | 1 | 49 | 21 | 39 |
| RA-60 | 10 | 7 | 1 | 5 | 15 | 34 | 8 | 38 | 30 | 50 |
| RV-20 | 26 | 19 | 14 | 31 | 52 | 53 | 49 | 45 | 48 | 50 |
| RV-40 | 14 | 12 | 3 | 7 | 6 | 26 | 1 | 49 | 15 | 40 |
| RV-60 | 10 | 7 | 1 | 3 | 15 | 34 | 11 | 38 | 31 | 50 |
| **Anatomical traits** |  |  |  |  |  |  |  |  |  |  |
| TCA | 29 | 3 | 17 | 26 | 14 | 5 | 7 | 30 | 53 | 1 |
| CCF | 28 | 4 | 7 | 26 | 17 | 1 | 2 | 40 | 46 | 11 |
| CCS | 19 | 46 | 52 | 8 | 16 | 44 | 47 | 6 | 40 | 37 |
| CCC | 30 | 3 | 9 | 25 | 17 | 2 | 4 | 50 | 43 | 12 |
| TSA | 29 | 50 | 51 | 33 | 36 | 40 | 49 | 21 | 48 | 20 |
| XVA | 36 | 39 | 46 | 8 | 27 | 13 | 40 | 2 | 51 | 4 |

For a particular trait, the ranking values presented (from 1^st^ to 53^th^) were based on the actual mean data for each trait with the lowest ranking (1^st^) being the least mean value among the 53 genotypes

**Table S3** List of the 53 alfalfa genotypes used in this study (41 Introduced cultivars, 9 Chinese breeding lines, and 3 Chinese native cultivars)

| Abbreviation | Genotypes | Origin | Characteristics |
| --- | --- | --- | --- |
| QT | Knight T | America | Fast regeneration rate, high productivity |
| DFH | Millionaire | Canada | Cold tolerant, fast regeneration rate,  high productivity |
| AND | Adrenalin | Canada | Disease resistant, fast regeneration rate |
| SD | Sardi 7 | Australia | Disease resistant, fast regeneration rate,  high productivity |
| BM | Blue moon | America | Hot tolerant, high productivity |
| CL | Central | Italy | Hot tolerant, high productivity |
| B310 | Bara 310 SC | America | Salt tolerant, rolling resistance |
| B218 | Bara 218 TR | America | Disease resistant, rolling resistance,  high productivity |
| PL | Paola | Italy | High quality |
| B420 | Bara 420 YQ | America | High quality, high productivity |
| B416 | Bara 416 WET | America | Humidity resistant, high productivity |
| QS2 | Knight No. 2 | America | Drought tolerant, cold tolerant,  fast regeneration rate |
| TZZ | Survivor | America | Cold tolerant, wind and sand resistant |
| DYH | Galaxie Max | France | Lodging resistant, high productivity |
| KS | Concept | America | Disease resistant |
| BB | Barricade | America | Salt tolerant |
| YS | Warrior | America | High productivity |
| WL319 | WL319HQ | America | Cold tolerant, high productivity |
| WL168 | WL168HQ | America | Drought tolerant, cold tolerant,  high productivity |
| WL298 | WL298HQ | America | Cold tolerant, salt tolerant, high productivity |
| WL354 | WL354HQ | America | Cold tolerant, disease resistant,  high productivity |
| WL363 | WL363HQ | America | Cold tolerant, high productivity |
| WL358 | WL358HQ | America | Cold tolerant, disease resistant,  high productivity |
| WL343 | WL343HQ | America | Cold tolerant, high productivity |
| WL366 | WL366HQ | America | Cold tolerant, salt tolerant, high productivity |
| Ca | Claudia | Italy | Disease resistant, lodging resistant,  fast regeneration rate |
| QJ | Vision | America | Lodging resistant, fast regeneration rate,  high productivity |
| LT | Instict | America | Cold tolerant, disease resistant |
| BJX | Gibraltar | America | Drought tolerant, cold tolerant,  barrenness resistant |
| XB | Xuebao | America | Cold tolerant |
| DY | DS310FY | America | Cold tolerant, disease resistant,  fast regeneration rate |
| MF | MF4020 | Canada | Cold tolerant, disease resistant,  high productivity |
| QTZ | Optimus | America | Disease resistant |
| SDL | Sanditi | France | Disease resistant, lodging resistant,  high productivity |
| PW | Power 4.2 | America | Disease resistant, high quality, high productivity |
| 3010 | SK3010 | Canada | Cold tolerant, disease resistant,  high productivity |
| LW | 6010 | Canada | Disease resistant, high productivity |
| JG | Aurora | Australia | Cold tolerant, fast regeneration rate |
| 5070 | 5070 | America | High quality, high productivity |
| MTW | Mattwi | America | Disease resistant, fast regeneration rate |
| LB | Lobo | America | Disease resistant, fast regeneration rate,  high productivity |
| G1 | Gannong No.1 | China | Drought tolerant, cold tolerant |
| G3 | Gannong No.3 | China | Drought tolerant, cold tolerant, barrenness resistant |
| G4 | Gannong No.4 | China | High productivity |
| Z1 | Zhongmu No.1 | China | Salt tolerant |
| Z2 | Zhongmu No.2 | China | Flooding tolerant |
| Z3 | Zhongmu No.3 | China | Salt tolerant |
| G6 | Gannong No.6 | China | High productivity |
| CY3 | Caoyuan No.3 | China | Cold tolerant, drought tolerant |
| L801 | Longmu No.801 | China | Cold tolerant, salt tolerant |
| AH | Aohan | China | Drought tolerant, cold tolerant |
| LD | Longdong | China | Drought tolerant |
| XJDY | Xinjiang Daye | China | Large leaf, fast regeneration rate |
